# Mixing and Thermal Transport Behavior in a Pin or Non-Pin Extruder Equipped with a Field Synergy Elongation Screw

**DOI:** 10.3390/polym16131793

**Published:** 2024-06-25

**Authors:** Yancai Sun, Shilong Wang, Shizheng Huang, Wei Pan, Yan He, Ranran Jian

**Affiliations:** College of Electromechanical Engineering, Qingdao University of Science and Technology, Qingdao 266061, China

**Keywords:** screw design, mixing, heat transfer, ductile extrusion, field synergy principle

## Abstract

The ductile forming process of a polymer in a standard screw extruder and pin-barrel extruder, equipped with or without a field synergy elongation screw, was investigated by the finite element method. In order to assess the mixing and heat transfer capabilities of screws, characteristic parameters such as the mixing efficiency, segregation scale, and temperature distribution of different structures were analyzed and compared. The results indicated that the flow pattern of the polymer melt in the extruder was significantly influenced by the screw structure and was improved by the newly designed field synergy screw configuration, which brought a desirable elongational flow to enhance the radial convection. This was attributed to the unique radial wedge-shaped repeated convergence region of the field synergy elongation screw, increasing the synergistic effect between the velocity field, velocity gradient field, and temperature gradient field and thus improving the heat transfer and mixing efficiency. After adding barrel pins, the flow was forced to split, resulting in a more significant stretching effect on the melt. The field synergy effect in the pin mixed region was strengthened, which further increased the heat and mass transfer efficiency of the screw. However, increasing barrel pins could also lead to undesired temperature fluctuation and flow resistance, which have a negative impact on the melt uniformity. This study offers an important reference for optimizing screw structure to obtain strong mixing and heat transfer performances.

## 1. Introduction

The extrusion technology, as a fundamental polymer processing technique, plays an important role in the preparation of polymers with tailorable properties, and the functional extruded products are applied in various fields, such as architecture, aerospace, electronic products, and medical devices. Currently, single screw extruders (SSEs) are widely used in the polymer extrusion industry due to their simple manufacturing operation and low production and maintenance costs. However, the mixing and thermal performances of a screw in an SSE directly affect the stability of the polymer’s subsequent processes and the quality of the end-product. Understanding the relationship between the temperature and velocity fields during the polymer ductile forming process driven by a screw is important for determining extrusion conditions, optimizing process parameters, improving screw design, and increasing production quality.

During the extrusion process, effective thermal and molecular mobility is essential to achieving good heat transfer and mixing, which is largely influenced by the screw structures. Therefore, in order to improve the production quality and reduce the production cost, various hybrid components employing pins, barriers, and specific flights have been developed and applied in SSEs. For example, Rauwendaal [1] designed and analyzed the Chris Rauwendaal dispersive (CRD)-type barrier screw with a slanted part on the top of the barrier flights, causing elongational flow in the high shear region to improve the melting and mixing performances of an extruder. Kimura et al. [2] conducted an analysis on a Dulmage-type distributive mixing screw, which contained several sets of Dulmage elements in the metering section of the screw tip, dividing the flow into multiple narrow channels along the axial region. Compared with the traditional single-threaded screw, a more uniform overall distributive mixing was obtained in the cross-section of the Dulmage-type screw. Pandey and Maia [3] developed an extended mixing element (EME) with fixed hyperbolic convergence-divergence channels to facilitate elongational flow, increasing the dispersion and mixing capabilities of screws in SSE. Liu et al. [4] studied the mixing properties of the wave screw element, as well as the barrier screw element, by numerical simulation using ANSYS POLYFLOW. Compared with the barrier screw, the wavy screw was found to be beneficial for the shear and stretch of the melt, resulting in a better mixing efficiency. He et al. [5] designed a mound-shaped extensional mixing element (M-EME) based on the geometric characteristics of sinusoidal curves to enhance stretching mixing, which increased the proportion of the stretching flow field in the mixing process, thereby affecting the mixing performance of the screw. Rauwendaal [6] developed an innovative screw structure that incorporated a split flow pattern, enabling the conversion of the melt between the central and surrounding areas and thus enhancing the mixing and heat transfer capabilities in the extruder. Wang et al. [7] performed a numerical analysis on the flow and heat transfer of a polymer melt in the screw channel for four different screw structures commonly used in extruders. The results showed that the screw structures were very important for material mixing and temperature uniformity during the extrusion process. Among a series of novel screw components, the pin-screw or pin-barrel, which force diversion, can increase stretch ductile deformation and redistribute the flow field, showing an excellent mixing capacity. The screw mixing progresses by repeating the dividing and combining flow in the pin zones, thereby improving the uniformity of the polymer melt. For instance, both Cheng et al. [8] and Wang et al. [9] found that installed pins on the barrel of the extruder increased the shear stress and stretch stress of the melt in the flow field, altering the particle trajectory of the melt and thereby improving the melting capacity and mixing uniformity of the extruder. The studies mentioned above indicated that designing and optimizing screw structures to control the flow pattern and ductile deformation can achieve the effective mixing and temperature uniformity of the melt.

In order to gain better understanding of the influence of screw structures on the fluid dynamics and thermodynamics of the melt during the polymer extrusion process, relevant researchers investigated and analyzed various characteristic parameters of screws. Bauer et al. [10] analyzed the residence time distribution in a silicon tracer experiment based on smooth particle hydrodynamics (SPH) and stated the mixing mechanism of five different screw structures. Connelly and Kokini [11] and Cheng et al. [12] analyzed the influence of structural factors on mixing during ductile extrusion, utilizing characteristic parameters such as the shear rate, stretching rate and mixing index to assess the mixing process of screw structures. Marschik et al. [13] used 3D non-Newtonian flow simulation to study the conveying and mixing capabilities of screws and provided a detailed introduction to the influence of screw geometry on conveying and mixing capabilities using theoretical parameters such as the pressure, mixing index, and shear stress. An et al. [14] investigated the three-dimensional flow field and dynamic mixing process in a continuous mixer using a quantitative evaluation system consisting of dispersed mixing and distributed mixing, demonstrating the influence of rotor structures on mixing performance in the continuous mixer. Grosso et al. [15] studied the stretching and folding mixing actions based on a simplified two-dimensional model of the cavity transfer mixer. According to the Poincaré maps and the evolution of fluid droplets, it was shown that the geometric structures of the mixing device had a certain impact on its distribution mixing. Kim [16] analyzed a range of parameters including the flow rate, pressure gradient, and helix angle to comprehensively understand and predict the residence time distribution (RTD) during single-screw extrusion. Yao et al. [17] calculated the distribution of the local mixing efficiency of a screw with a pin mixing section using fluid mixing kinematics theory and provided a quantitative assessment of the overall mixing capacity of the equipment. Xu et al. [18] proposed a new mapping method that converted the transient flow field at an initial time into a velocity field at any time and investigated the influence of screw speed on distribution mixing. Lim et al. [19] introduced a concept of partial rotation fraction within the periodic unit of helical geometry in the metering zone of an SSE, aiming to comprehend and predict the screw characteristics. On the basis of the in-depth analysis of conservation equations in continuum mechanics, Jian et al. [20] incorporated the multiple field synergy concept into polymer processing and proposed the polymeric field synergy principle. Emphasis was placed on the synergy between the velocity field and temperature gradient, as well as between the velocity field and velocity gradient in the flow process, and a series of novelty screw configurations were designed to achieve both mixing and heat transfer enhancement.

In this study, we proposed a novel screw, called a field synergy elongation screw, designed based on the polymeric field synergy principle. The mixing and thermal transport process of a polymer melt in different screw structures was compared and analyzed by calculating the three-dimensional non-isothermal flow field. The changes in the velocity and temperature fields of the newly designed field synergy elongation screw with or without barrel pins were analyzed by tracing the particle distribution, field synergy analysis, and other characteristic parameters. Our aim is to demonstrate the mixing and heat transfer performances of the new field synergy elongation screw during the ductile forming process of a polymer in a pin or non-pin extruder and to provide a positive prospect for optimizing the screw structural design of extruders.

## 2. Polymeric Field Synergy Principle

In the case of a steady laminar flow and thermal transport process in the polymer processing, we have simplified the momentum and energy conservation equations into the following dimensionless forms:

(1) $$Re\left(\bar{V}\cdot \nabla \bar{V}\right)+\pi_{P}=\nabla \cdot \bar{\tau }$$

(2) $$Re\;Pr\int_{0}^{1}\left(\bar{V}\cdot \bar{\nabla T}\right)d\bar{r}-\overset{\cdot }{\Phi }=Nu$$

where $\bar{V}\;$ is the velocity vector, $\bar{\nabla V}$ is the velocity gradient vector, $\bar{\nabla T}$ is the temperature gradient vector, $\bar{\tau }$ is the stress tensor, $\bar{r}$ is the r component in a cylindrical coordinate, $\overset{\cdot }{\Phi }$ is the viscous dissipation energy, *Re* is the Reynolds number, *π_P_* is the dimensionless pressure gradient, *Pr* is the Prandtl number, and *Nu* is the Nusselt number. *Re*, *π_P_*, *Pr*, and *Nu* are expressed as

*Re* = *ρv_f_δ*/*η_f_*, *π_P_* = ∇*Pδ*/*τ_f_*, *Pr* = *C_P_ η_f_*/*λ_f_*, *Nu* = *hδ*/*λ_f_* (3)

where *ρ* refers to the fluid density, *v_f_* refers to the fluid average velocity, *δ* refers to the feature dimension, *η_f_* refers to the fluid average viscosity, *P* refers to the pressure, *τ_f_* refers to the fluid average stress, *C_P_* refers to the constant-pressure specific heat, *λ_f_* refers to the fluid average thermal conductivity, and *h* refers to the heat convection coefficient.

Equation (1) reveals that the fluid momentum variation, affecting the momentum transport and viscous stress $\bar{\tau }$ among fluid particles, is dependent on the interaction between the velocity and velocity gradient fields. In fact, viscous stress is the main factor that causes fluid to stretch or shear in processing to affect the momentum and mixing (mass transfer) of a polymer melt. Therefore, stretch or shear ductile deformation is highly correlated with the interaction of the velocity and velocity gradient fields. The dot product in Equation (1) can be described as

(4) $$\bar{V}\cdot \bar{\nabla V}=\left|\bar{V}\right|\left|\bar{\nabla V}\right|\cos\;\alpha$$

where *α* is the included angle between the velocity vector and the velocity gradient vector and can be expressed as Equation (5)

(5) $$\alpha =\cos^{-1}\left(\frac{\bar{V}\cdot \bar{\nabla V}}{\left|\bar{V}\right|\left|\bar{\nabla V}\right|}\right)$$

Here, *α* is defined as the synergy angle between the velocity vector and velocity gradient, which represents the synergy capacity between the velocity and velocity gradient. In the range 0–90°, Equation (4) increases with decreasing *α*, which improves the momentum transport and viscous stress. Interestingly, the synergy angle *α* equals zero, the flow field is pure extensional flow, and yet the synergy angle α becomes 90°, and pure shear flow occurs. As we know, elongational ductile flow is more conducive to mixing than shear flow in polymer processing. Apparently, field synergy analysis fundamentally clarifies the mixing mechanism of the elongational flow in polymer processing. Consequently, if the direction of the fluid velocity tends closer to that of the velocity gradient, to bring more elongational flow and to increase the viscous stress, the mixing and momentum transport will be better in the laminar flow field.

Similarly, Equation (2) shows that the heat transfer is dependent on the Reynolds number, the Prandtl number, and the integral of the velocity and temperature gradient if the viscous dissipation energy is constant. This equation suggests a new way to enhance heat transfer by increasing the integral. The dot product in the integral in Equation (2) can be described as

(6) $$\bar{V}\cdot \bar{\nabla T}=\left|\bar{V}\right|\left|\bar{\nabla T}\right|\cos\;\beta$$

where *β* is the included angle between the velocity vector and the temperature gradient vector and can be expressed as Equation (7)

(7) $$\beta =\cos^{-1}\left(\frac{\bar{V}\cdot \bar{\nabla T}}{\left|\bar{V}\right|\left|\bar{\nabla T}\right|}\right)$$

Here, *β* is defined as the synergy angle between the velocity vector and temperature gradient, which represents the synergy capacity between the velocity and temperature gradient. In the range 0–90°, Equation (6) increases with decreasing *β*, which improves the thermal transport and convective heat transfer between the fluid and the solid wall. That is, if the direction of fluid velocity tends closer to that of heat flux, heat transfer will be better in the laminar flow field. This polymeric field synergy principle provides a novel perspective for understanding the behavior of mixing and thermal transport in polymer processing and offers a new way to enhance these behaviors.

## 3. Mathematical and Physical Models

**Figure 1:**
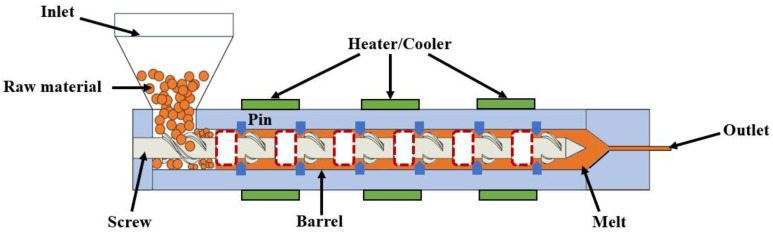
Extrusion process of the polymer in the SSE.

The illustration of a pin-barrel extruder model was depicted in Figure 1. The extrusion process involved the addition of polymer raw material to the feed port of the extruder; then, the raw material was pushed through the screw to the mixed melting area of the extruder. In this process, polymer raw material was heated to its melting point and transformed into a molten state through barrel heating or screw mechanical action. Once the polymer reached a completely molten state, it was extruded through the outlet of the extruder. To investigate the impact of barrel pins on the mixing and heat transfer performances in the extruder, a series of pins were incorporated into the barrel at 60 mm intervals, starting from the 30 mm position of the screw tip. Each row consisted of six pins evenly distributed along the circumference.

### 3.1. Physical Model

**Figure 2:**
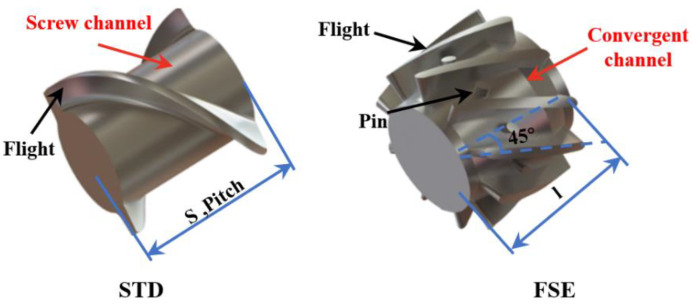
The screw elements.

The geometric configurations of the screw elements used in the present work are illustrated in Figure 2. One of these components was the standard double-threaded (STD) element in the industry, and the other was the new field synergy elongation (FSE) element with a working length of 24 mm and a flight angle of 45°. The FSE element had ten flights uniformly distributed in the circumferential direction, forming ten identical regions with a radial wedge-shaped convergent channel, described as the stretching flow region. Each region comprised a stretching upward slope equipped with a pin and a stretching downward slope. In these regions, a highly viscous polymer was expected to generate elongational deformation, thus stretching the melt into a thin layer and increasing the specific surface area for heat transfer enhancement, ultimately enhancing both mixing and temperature uniformity.

**Figure 3:**
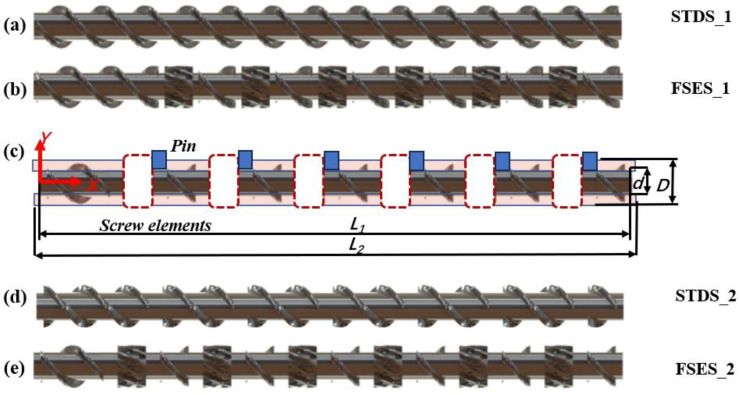
The dimensional model used in the simulation: (**a**) STDS_1; (**b**) FSES_1; (**c**) flow domain model; (**d**) STDS_2; (**e**) FSES_2.

The geometric structures of the screws between the two groups with or without pins in this study are illustrated in Figure 3. Among them, Figure 3a,d depict the standard double-threaded screws (STDS_1 and STDS_2) commonly used in industry; Figure 3b,e depict the newly designed field synergy elongation screws (FSES_1 and FSES_2), which incorporate six sets of FSE elements into an STD screw. Figure 3c depicts the flow domain models of STDS_2 and FSES_2 after being fitted with barrel pins. To prevent dimensional interference between pins and screw fights, the distance of each of the two components was increased with an extra transitional polish rod. In order to mitigate the impact of entrance and export effects on the calculation, the flow region was extended outward by 2 mm in the boundary of the inlet and outlet respectively. The pin had a diameter of 6 mm and a height of 4.5 mm, and other geometric parameters are detailed in Table 1.

### 3.2. Governing Equations and Boundary Conditions

Polymer extrusion is a complex flow process that involves the interaction of multiple factors such as viscous laminar flow, screw driven motion, viscous heating, ductile deformation, phase transition, and heat transfer. In this study, ethylene propylene diene monomer (EPDM) was chosen as the working fluid under non-isothermal transient flow. The fluid was assumed to be incompressible and was completely filled in the flow domain, with no slip on the wall. The viscosity of a polymer melt is usually very high; thus, the Reynolds number of the melt flow in the extruder is much smaller than one, so the influence of inertia can be ignored. Under the above assumptions, the flow field followed the continuum mechanics equations: mass conservation, momentum conservation, and energy conservation.

Continuity equation:

(8) $$\frac{\partial u_{i}}{\partial x_{i}}=0$$

Momentum equation:

(9) $$\rho \frac{\partial ui}{\partial t}+\frac{\partial p}{\partial xi}=\frac{\partial }{\partial x_{j}}(\eta \frac{\partial u_{i}}{\partial x_{j}})$$

Energy equation:

(10) $$\rho C_{P}\left(\frac{\partial T}{\partial t}+u_{i}\frac{\partial T}{\partial x_{i}}\right)=k\frac{\partial^{2}T}{\partial x_{i}^{2}}+\varphi$$

where *u_i_* is the *i* component of velocity, *x_i_* is the *i* component of the position in a Cartesian coordinate, *t* is the time, and *T* is the temperature.

The Bird–Carreau and Approximate Arrhenius laws were used to characterize the rheological properties of the EPDM material. $\overset{\cdot }{\gamma }$ is the shear rate. The material properties of EPDM are shown in Table 2, and the model boundary conditions are listed in Table 3.

(11) $$\eta =[\eta_{\infty }+(\eta_{0}-\eta_{\infty })(1+\lambda^{2}{\overset{\cdot }{\gamma }}^{2}{)}^{\frac{n-1}{2}}]exp[-f(T-T_{\alpha })]$$

### 3.3. Mesh System and Independence Validation

ANSYS Polyflow 17.0 (ANSYS, Inc., Canonsburg, PA, USA) computational fluid dynamics (CFD) software was employed in the calculation, and ANSYS ICEM 17.0 mesh software (ANSYS, Inc., Canonsburg, PA, USA) was used to generate the mesh system. Among them, the hexahedral structure network was used to discretize the flow domain, and the boundary layer near the barrel wall was refined to achieve the accurate data of small gaps between the barrel and screw flight. An unstructured network of tetrahedral elements was established for screws because of the irregular structure. The mesh super-position technique (MST) was used between the flow region and the moving screw to accommodate changes in the boundary grid over screw rotation.

**Figure 4:**
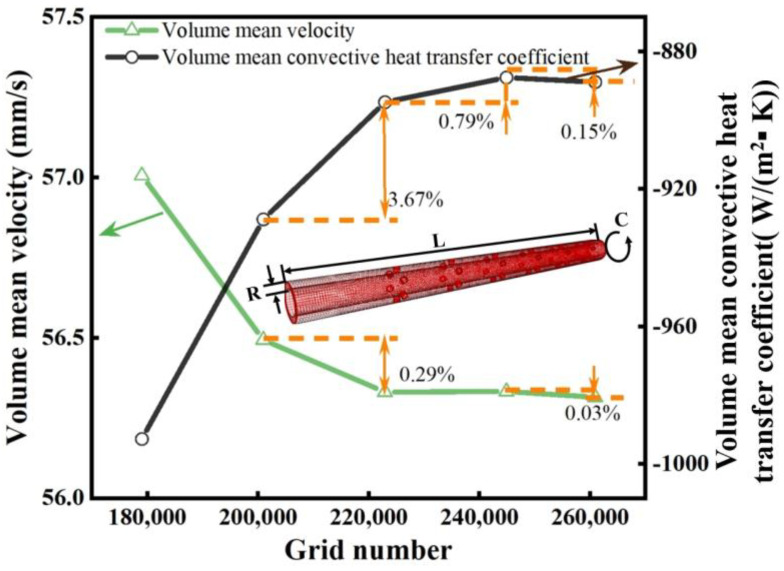
Mesh independence validation of the fluid model.

Figure 4 shows the grid independence analysis from the three dimensions of radial (R), axial (L), and circumferential (C) to ensure the reliability of the calculation result. As depicted in Figure 4, when the total number of grids was about 220,000, the variations for the volumetric mean velocity and volume mean convective heat transfer coefficient were less than 1%. Therefore, considering the balance between computational efficiency and accuracy, a mesh layout system of 18 × 70 × 160 (R × C × L) will be adopted in subsequent numerical simulations, with a total number of grids of 222,920.

## 4. Result and Discussion

### 4.1. Flow Filed

**Figure 5:**
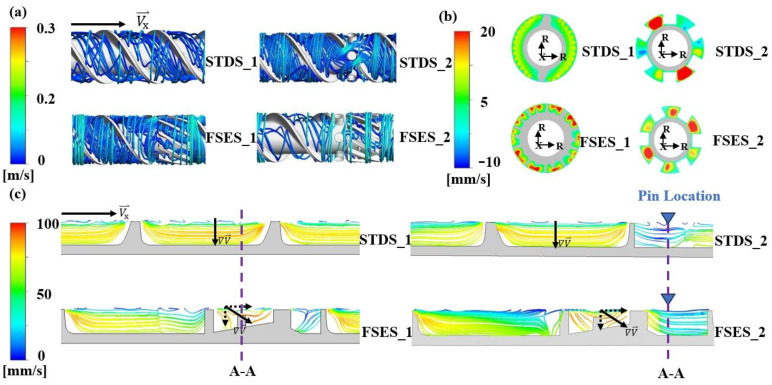
Flow analysis for various screws: (**a**) velocity streamlines in the axial direction; (**b**) axial velocity contours in the cross-section A-A; (**c**) axial velocity streamlines in screw channels.

Figure 5 illustrates the flow field in each screw channel at a speed of 60 r/min. As depicted in Figure 5a,c, the flow of the melt in the STDS_1 structure was predominantly characterized by a steady laminar flow, with almost no radial flow. Unlike the flow pattern in a standard screw, the melt in FSES_1 generated a distinct spiral streamline when passing through the channels of the FSE element, as shown in Figure 5a. Figure 5b plots the impact of screw structures on the radial velocity distribution. Compared to the STDS_1, the FSES_1 was affected by the wedge-shaped convergence region of the FSE element, resulting in an increase in velocity and the mutual compression and stretching of melt layers. The melt was transported forward in a more complex stretching flow pattern through the radial wedge-shaped convergent channels of the FSE element, since the velocity gradient increased along the axial direction, as shown in Figure 5c. The melt layer was thinned, gradually passing through the radial wedge-shaped convergent channels, and reached its peak at the highest point of the slope. The unique wedge-shaped channel was beneficial for the melt to deviate from the laminar flow direction and reorient to enhance the heat and mass transfer process. Furthermore, the addition of barrel pins had a significant impact on the flow pattern of the melt in STDS_2 and FSES_2. The pin structure forced the melt to split, bringing the stable laminar flow to a more irregular state.

### 4.2. Pressure Difference and Conveying Capacity

**Figure 6:**
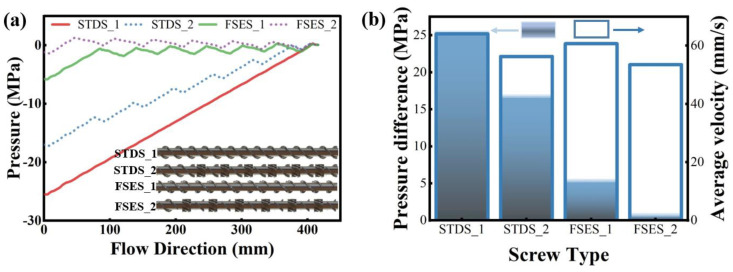
Flow pressure distribution (**a**) and pressure difference and flow velocity (**b**) in various screws.

Figure 6 illustrates the pressure performance and flow velocity of the studied screws. From the pressure distribution curves of the melt at a speed of 60 r/min in Figure 6a, we found that the FSES_1 exhibited a significant pressure step change along the axial direction compared with STDS_1. A similar phenomenon was also observed in the screws equipped with pins. Neither the pins or FSE elements affect the pressure building ability, resulting in a pressure drop in the location of elements and lower inlet and outlet pressure differences, as shown in Figure 6b. The presence of the FSE elements and barrel pins in screws formed complex screw channels and might cause flow resistance. However, the frequent alternation of high and low pressure in the axial direction of the screw also caused the melt to undergo repeated physical effects such as compression, stretching, relaxation, alternation, and displacement, improving the stretching effect of screw configurations on the polymer melt to promote mixing.

In order to further analyze the impact of screw configurations on the melt conveying performance, we calculated the volume average velocity, as shown in Figure 6b. The average velocity of FSES_1 was slightly lower than that of STDS_1, but the average velocity of both sets of screws decreased obviously after the addition of barrel pins. It can be concluded that pins could significantly reduce the flow rate and increase the flow resistance. Although the FSE and pin structures caused additional pressure resistance to a certain extent, they also increased the residence time of particles in the fluid channels, which could make the melt mix more thoroughly.

### 4.3. Particle Trajectories

**Figure 7:**
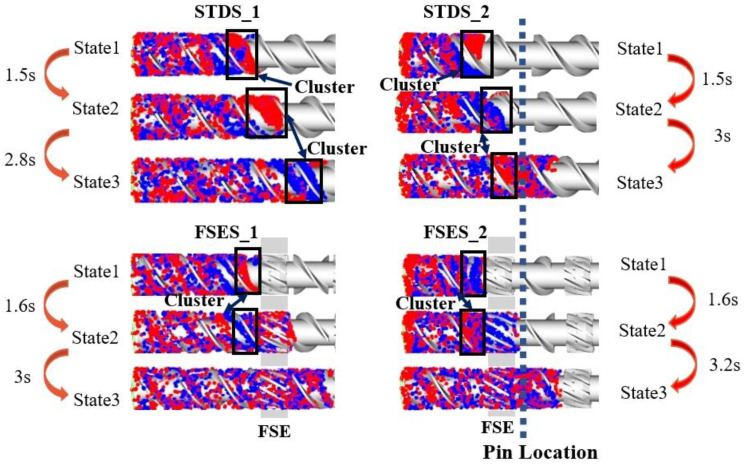
Tracer particle analysis of the screw at different time states.

To further investigate the complex flow patterns of melt particles in various screw structures and their impact on mixing and heat transfer capabilities, we employed the tracer particle motion method to analyze and track the movement, mixing effect, and residence time distribution of fluid particles. Figure 7 illustrates the mixing process of tracer particles in screw channels at various time states. Initially, the tracer was randomly placed at the inlet of the flow field, with two colors of each accounting for half. Next, the movement of the tracer was captured by the variation in screw motion. It can be observed that as the state evolved over time, particles gradually intermingled with each other. For the comparison of different screw elements, State 1 of the particle was denoted as the location in the inlet of the FSE element; State 2 was the location in the outlet of the FSE element before pins; and State 3 was the location after flowing out of the pin regions to the next FSE element. The time interval between adjacent states was defined as the residence time of the particles.

In Figure 7, it can be seen that the tracer was transported along the fluid channel divided by the STD element before the polymer melt passed through the FSE element and barrel pins. In STDS_1, the phenomenon of particle cluster still existed significantly with an increase in flow time. This can be attributed to the limitation of its structure, which made the melt in a simple laminar flow that inhibited uniform particle mixing. In FSES_1, the FSE configuration divided the channel into several relatively small stretching convergence regions to undergo complex movements such as compression, stretching, and spiral displacement, resulting in a significant improvement in the particle mixing performance. The pins also forced the melt to divide and combine repetitively and thus enhanced mixing. Therefore, in STDS_2 and FSES_2, the cluster of particles after flowing pass pins was reduced. It can be concluded that the addition of pins further improved the mixing performance of the screws.

We further calculated the residence time taken for the tracers to reach each state in various screws, as illustrated in Figure 7. Compared to STDS_1, STDS_2, and FSES_1, we found that both the barrel pins and the new FSE element extended the residence time of particles in the screw mixing section. FSES_2 also proved this point well, which had the maximum residence time (4.8 s from State 1 to State 3) among these screws. The results indicated that the field synergy elongation element effectively extended the residence time of the polymer melt in the mixing section and prolonged the ductile forming history of the screw on the polymer melt, thus improving the mixing ability of the screw.

### 4.4. Mixing Characteristics

**Figure 8:**
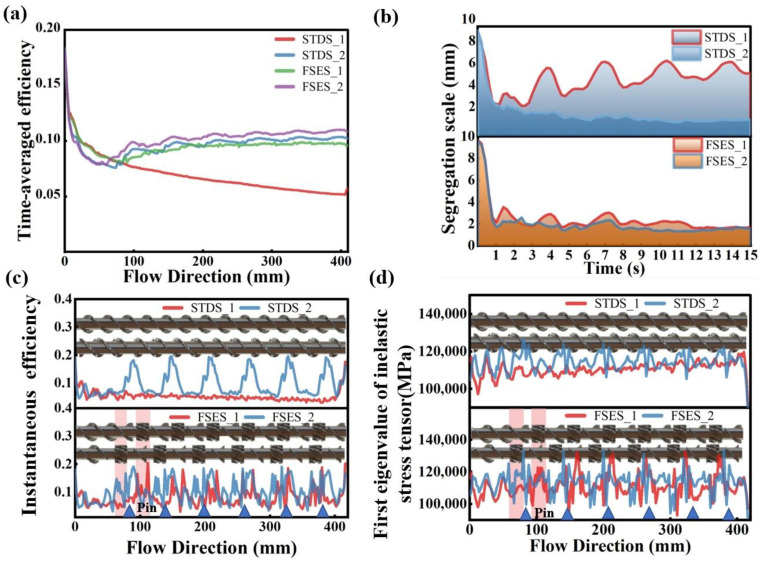
Mixing properties of various screws: (**a**) time-averaged mixing efficiency; (**b**) segregation scale; (**c**) instantaneous mixing efficiency; (**d**) first eigenvalue of the inelastic stress tensor.

Figure 8 presents a range of common indicators for evaluating mixing capacity, among which time-averaged efficiency measures the capability of flow deformation and interface generation to assess the overall mixing effectiveness of the screw. As shown in Figure 8a, the order of time-averaged efficiency was FSES_2 > STDS_2 > FSES_1 > STDS_1. It is noteworthy that the data of STDS_1 were much lower than those of the other three. The results suggested that the FSE element was capable of significantly enhancing mixing. In FSES_2, the time-averaged mixing efficiency was further increased by the pins, from 0.09 in FSES_1 to 0.11, resulting in a 22.2% improvement.

The segregation scale reflects the uniformity of the particle concentration in the mixing state, and small values of the segregation scale imply uniform mixing. As depicted in Figure 8b, it can be found that the FSES_2 exhibited the lowest segregation scale among the two sets of screws, while the STDS_1 demonstrated the highest segregation scale. The addition of pins further decreased the segregation scale to promote a more uniform mixing of fluids, ultimately reducing the non-uniformity between melts. These findings suggested that the mixing uniformity of the STDS_1 was suboptimal, whereas both the FSE element and the pins contributed to an improvement in melt mixing uniformity to a certain extent. Furthermore, the segregation scale of the FSES_1 was relatively stable, with a small numerical difference compared with the conventional STDS_1.

Similarly, the distribution of instantaneous mixing efficiency along the axial direction and the first eigenvalue of the inelastic stress tensor revealed similar results. Instantaneous mixing efficiency is an efficiency indicator for a specific moment and the inelastic stress tensor is the core stress to break drops. Figure 8c,d demonstrated a significant increase in instantaneous mixing efficiency and the first eigenvalue of the inelastic stress tensor at the positions of both the FSE element and pins. With the combination of the FSE configuration and pins, an excellent mixing ability could be achieved.

### 4.5. Temperature Uniformity

**Figure 9:**
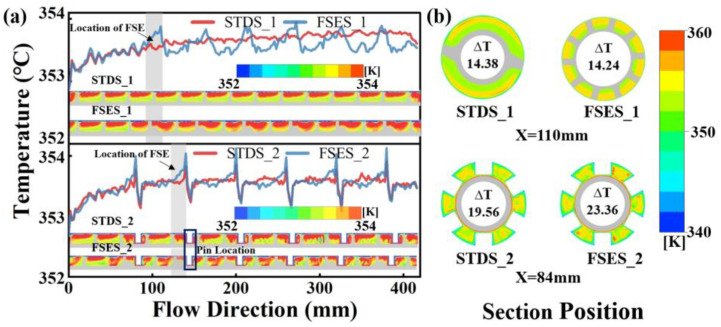
Temperature performance: (**a**) axial temperature distribution; (**b**) radial temperature distribution.

Figure 9 plots the axial temperature distribution of screws and the radial temperature difference between two sets of screws at different axial positions. As shown in Figure 9a, when the melt flowed into the FSE channels in FSES_1, the temperature of the melt rose first and then gradually stabilized as the melt flowed out of the element, and it was lower than that in STDS_1. The analysis indicated that when the polymer melt initially entered the FSE element, the temperature increased due to the viscous heat generation; subsequently, as a result of the heat transfer and mixing effects, the temperature field tended to stabilize with a lower temperature than those observed in standard screws. The radial heat transfer capability of the FSE element surpassed that of the standard screws. The axial temperature fluctuation increased violently in the zones of barrel pins. However, after passing through the mixing area, the temperature in FSES_2 was still lower than that in STDS_2, and the curve tended to stabilize. As depicted in Figure 9b, the impact of screw configurations on the melt temperature uniformity was further investigated by selecting and observing the temperature contours of each screw at the cross-section of 84 mm and 110 mm away from the inlet. The FSES_1 exhibited a uniform radial temperature distribution, since the radial temperature difference was lower compared to that of the STDS_1. After adding the barrel pins, both the standard screw and the field synergy elongation screw showed a significant increase in radial temperature fluctuations. The pins, to some extent, increased the local heating and had a negative impact on the temperature uniformity.

### 4.6. Field Synergy Analysis

**Figure 10:**
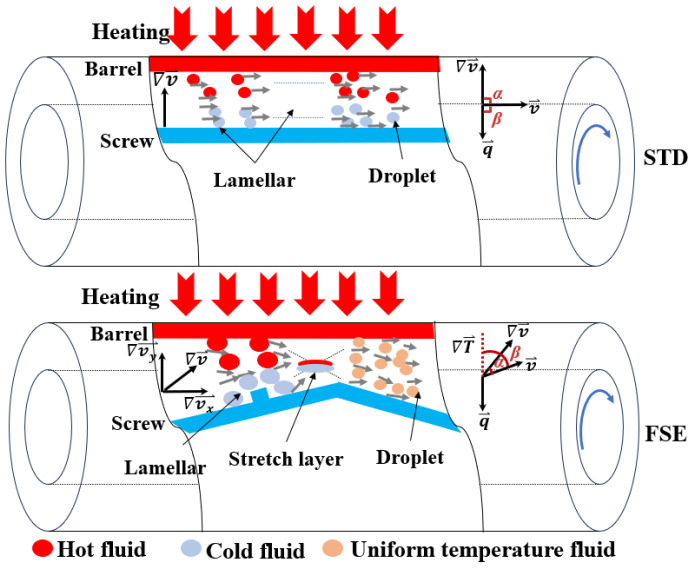
The synergistic phenomenon of polymer flow and heat transfer ($\overset{⃑}{q}$ is the heat flux).

In order to reveal the mechanism of mixing and heat transfer, field synergy analysis of the two screw configurations with different flow patterns was carried out. Figure 10 illustrates the heat and mass transfer behaviors of the melt in the flow channels. In the STD channel, the flow is laminar with smooth flow between layers, resulting in almost no radial motion between the bottom and the top of the screw channel; thus, the velocity gradient and temperature gradient are along the radial direction, i.e., their vector directions are perpendicular to the direction of velocity. In the FSE channel, the wedge-shaped region disrupts the inherent laminar flow pattern of the standard screw, generating a spiral–elongational flow with positive radial motion. This disruption causes radial displacement of the melt along the flow direction and changes the vectors interrelationship of the velocity, velocity gradient, and temperature gradient. According to the polymeric field synergy principle, a small field synergy angle represents good synergy effect for enhancing the mixing and heat transfer capacities of a screw.

**Figure 11:**
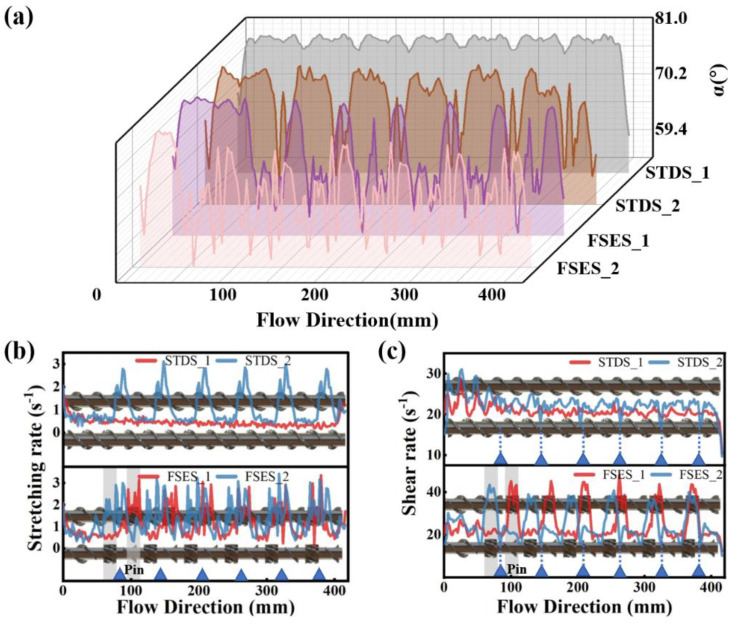
Mixing synergy and ductile deformation for screws: (**a**) synergy angle *α* along the flow direction; (**b**) shear rate distribution; (**c**) stretching rate distribution.

Figure 11a shows the distribution of the synergy angle *α* between the velocity and velocity gradient. It is evident that the synergy angle *α* of various screws follows the following order: FSES_2 < FSES_1 < STDS_2 < STDS_1. The results indicated that the significant elongational flow occurred in the FSE screw, which was consistent with the results in Figure 5, and the pins also caused elongational flow in channels, as further evidenced by the distribution of the stretching rate in Figure 11b. After being combined with the barrel pins, the proportion of stretching in FSES_2 further increased, greatly improving the mixing ability. The unique radial wedge-shaped convergent channels of the FSE element reduced the included angle between the velocity vector and the velocity gradient vector, causing the significant elongational ductile deformation of the polymer melt. For the shear rate of the different screws shown in Figure 11c, it can be found that the shear rate increased in the positions of the FSE element but decreased in the positions of the barrel pins. In conclusion, compared with the STD configuration, the stretching and shear rates of the FSE configuration were significantly improved, thus effectively enhancing the mixing performance. The barrel pins further facilitated the stretching deformation of the melt.

**Figure 12:**
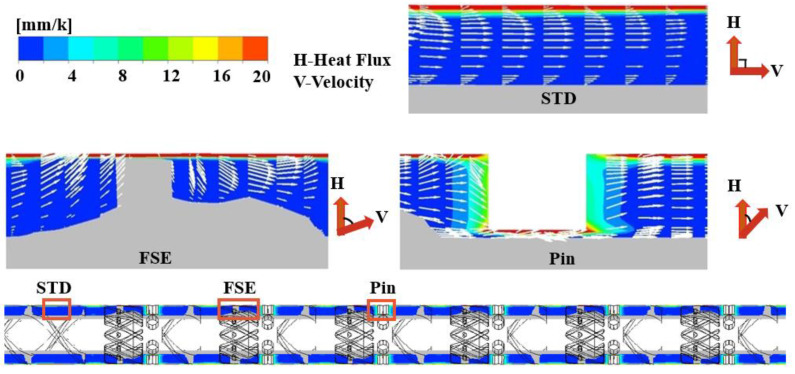
The temperature gradient contours and axial velocity vector in different screw channels.

As illustrated in Figure 12, to investigate the heat transfer enhancement mechanism of the FSE element and barrel pins on the radial direction of the screw, we conducted an analysis of the variation between the velocity vector and temperature field. In the STD channel, the polymer melt moved axially in laminar flow with almost no radial motion, and the velocity vector was nearly parallel to the direction of the flow and isotherm. The radial wedge-shaped convergent channel in the FSE element caused the flow direction of the melt to deviate, changing its velocity direction, which was conducive to heat exchange between the melt and the barrel wall. At the barrel pin, the flow pattern of the polymer melt became increasingly complex. In certain regions along both the radial and circumferential directions, the angle between the velocity field and temperature gradient field approached 0°.

**Figure 13:**
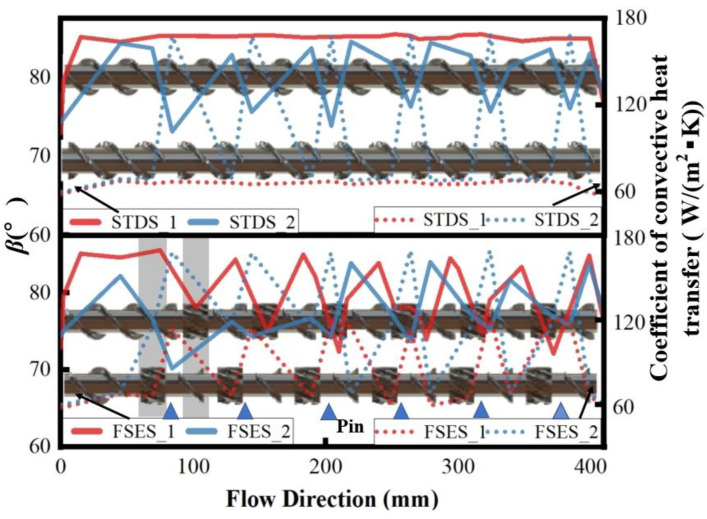
Synergy angle *β* and coefficient of convective heat transfer along the flow direction.

The convective heat transfer coefficient measures the heat transfer capability between the fluid and the wall, which is closely associated with the screw configuration and flow condition. Figure 13 shows the variation in the convective heat transfer coefficient and synergy angle *β* along the flow direction. It can be seen that there was a significant increase in the convective heat transfer coefficient and a significant decrease in the synergy angle *β* between the velocity and temperature gradient at the FSE and pin locations, which was consistent with the results in Figure 12. Overall, the convective heat transfer coefficient was strictly negatively correlated with the synergy angle *β*, and the smaller the synergy angle, the higher the convective heat transfer coefficient. The synergy angle *β* of each screw followed the following order: FSES_2 < FSES_1 < STDS_2 < STDS_1. The FSE element improved the synergy between the velocity vector and temperature gradient and increased the heat transfer between the polymer melt and the barrel wall. After adding barrel pins, the heat transfers of FSES_2 and STDS_2 further increased.

## 5. Conclusions

The flow and thermal transport behaviors of a polymer melt in conventional and pin-barrel extruders equipped with a field synergy elongation screw were studied by the computational fluid dynamics method based on the polymeric field synergy principle. The conclusions are as follows:

(1) Screw structures had a significant influence on the flow pattern and ductile deformation. For the STD configuration, only simple laminar flow was observed. The FSE configuration generated elongational flow in the mixing region, causing the mutual squeezing and thinning of the polymer melt, and facilitated the dispersion and dynamic mixing of aggregates. After being combined with barrel pins, the stretching effect was further improved with a long residence time and large mixing efficiency and thus increased the mixing ability of the entire plasticizing extrusion system.
(2) Due to the unique radial wedge-shaped convergent channel, the heat and mass transport were enhanced in the FSE element, accelerating the heat transfer and mixing. As a result, the temperature uniformity of the polymer melt during extrusion was improved. However, significant temperature fluctuations and viscous heat generation occurred at the pin locations, potentially compromising the overall temperature uniformity of the polymer melt.
(3) The field synergy principle revealed the mechanism of mixing and heat transfer. Compared with the conventional screw, the radial wedge-shaped convergence region of the FSE screw caused a significant radial deviation of the velocity vector in the channel, leading to an improved synergistic effect between the fields of velocity, velocity gradient, and temperature gradient and thus increasing the stretching stress and heat transfer efficiency. Barrel pins further increased the synergistic effect between various physical fields.

This study demonstrated that the unique field synergy elongation screw had a good performance in enhancing mixing and heat transfer capabilities and led to a certain improvement in temperature uniformity during the ductile extrusion process. Upon the inclusion of barrel pins, the mixing and heat transfer behaviors of the screw further enhanced, causing local temperature fluctuations in the melt. This provides a positive prospect for the structural optimization of a pin extruder equipped with a field synergy elongation screw.

## Figures and Tables

**Table 1:** Geometric parameters of the screw model.

Parameters	Symbol	Value (mm)
Screw length	*L*_1_	414
Screw outer diameter	*D*	30
Screw inner diameter	*d*	20
Flow domain length	*L*_2_	418

**Table 2:** Properties of EPDM employed in the calculation.

Parameter	Symbol	Value
Density	*ρ*	1150 Kg/m^3^
Specific heat capacity	*C_p_*	1600 J/(Kg·K)
Thermal conductivity	*k*	0.25 W/(m·K)
Viscosity at an infinite shear rate	*η_∞_*	0 Pa·s
Zero shear viscosity	*η*_0_	175,000 Pa·s
Natural time	*λ*	14 s
Non-Newtonian index	*n*	0.408
Coefficient of temperature sensibility	*f*	0.0025 K^−1^
Reference temperature	*T_α_*	373.15 K

**Table 3:** Boundary conditions.

Boundary	Flow Conditions	Thermal Conditions
Inlet	Fully developed, Volumetric flow rate 2.44 × 10^−6^ m^3^/s	353.15 K (80 °C)
Outlet	Flow Outflow	Heat outflow
Barrel wall	Stationary, No-slip wall	343.15 K (70 °C)
Screw wall	Screw speed 60 r/min	Insulated boundary

## Data Availability

The data presented in this study are available on request from the corresponding author.

## Nomenclature

*Re*	Reynolds number
$\bar{V}$	velocity vector, m/s
$\overset{⃑}{v}$	fluid velocity vector, m/s
$\bar{\nabla v}$	velocity gradient vector, s^−1^
$\bar{\nabla T}$	temperature gradient vector, K/m
$\bar{\tau }$	stress tensor, Pa
$\overset{\cdot }{\Phi }$	viscous dissipation energy
*π_P_*	dimensionless pressure gradient
$\bar{\tau }$	stress tensor, Pa
*Pr*	Prandtl number
*Nu*	Nusselt number
*ρ*	density, kg/m^3^
*v_f_*	fluid average velocity, m/s
*δ*	feature dimension, mm
*η_f_*	fluid average viscosity
*P*	pressure, Pa
*τ_f_*	fluid average stress, Pa
*C_p_*	constant-pressure specific heat capacity, J/(kg⋅K)
*λ_f_*	fluid average thermal conductivity, W/m·K
*h*	heat convection coefficient, W/(m^2^·K)
*α*	interaction angle between velocity and velocity gradient, °
*β*	interaction angle between velocity and temperature gradient, °
*L*_1_	screw length, mm
*D*	screw outer diameter, mm
*d*	screw inner diameter, mm
*L*_2_	flow domain length, mm
*k*	thermal conductivity, W/(m·K)
*η_∞_*	viscosity at an infinite shear rate, Pa·s
*η*_0_	zero shear viscosity, Pa·s
*λ*	natural time, s
*n*	non-Newtonian index
*f*	coefficient of temperature sensibility, K^−1^
*T_α_*	reference temperature, K

## References

[1] Rauwendaal C. (2005). Recent advances in barrier screw design. Plast. Addit. Compd..

[2] Kimura K., Nakayama Y., Kajiwara T. (2021). Distributive mixing characteristics of a Dulmage-type screw for a single-screw extruder: Experimental and numerical evaluation. Chem. Eng. J. Adv..

[3] Pandey V., Maia J.M. (2021). Extension-dominated improved dispersive mixing in single-screw extrusion. Part 1: Computational and experimental validation. J. Appl. Polym. Sci..

[4] Liu T.-L., Du Y.-X., He X.-Y. (2023). Statistical research on the mixing properties of wave based screws by numerical simulations. Int. Polym. Process..

[5] He H., Li W., Huang Z., Tian G., Zhu Z. (2023). Numerical analysis of a new mound-shaped extensional mixing element designed based on a sine curve in single-screw extrusion. Int. Polym. Process..

[6] Rauwendaal C. (2004). New screw design for cooling extruders. Plast. Rubber Compos..

[7] Wang C., Bussmann M., Park C. (2010). Numerical investigation of the effect of screw geometry on the mixing of a viscous polymer melt. J. Appl. Polym. Sci..

[8] Chen J., Dai P., Yao H., Chan T. (2011). Numerical analysis of mixing performance of mixing section in pin-barrel single-screw extruder. J. Polym. Eng..

[9] Wang Z., Pan Y., Liu Y., Huang J., Wang N., Hu X. (2022). Investigations of the Elongational Deformation Induced by Pins in Pin-Barrel Cold-Feed Extruders. Adv. Polym. Technol..

[10] Bauer H., Matić J., Evans R.C., Gryczke A., Ketterhagen W., Sinha K., Khinast J. (2022). Determining local residence time distributions in twin-screw extruder elements via smoothed particle hydrodynamics. Chem. Eng. Sci..

[11] Connelly R.K., Kokini J.L. (2007). Examination of the mixing ability of single and twin screw mixers using 2D finite element method simulation with particle tracking. J. Food Eng..

[12] Cheng W., Xin S., Chen S., Zhang X., Chen W., Wang J., Feng L. (2021). Hydrodynamics and mixing process in a horizontal self-cleaning opposite-rotating twin-shaft kneader. Chem. Eng. Sci..

[13] Marschik C., Osswald T.A., Roland W., Albrecht H., Skrabala O., Miethlinger J. (2019). Numerical analysis of mixing in block-head mixing screws. Polym. Eng. Sci..

[14] An Y., Yang W.M., Ding Y.M. (2012). Numerical Investigation of the Effect of Rotors’ Geometric Structure on Mixing Properties of Continuous Mixer. Key Eng. Mater..

[15] Grosso G., Hulsen M.A., Overend A., Anderson P.D. (2018). Fluid flow and distributive mixing analysis in the cavity transfer mixer. Macromol. Theory Simul..

[16] Kim S.J. (2018). Dimensionless analysis of three-dimensional residence time distribution in single-screw extrusion processes. Korea-Aust. Rheol. J..

[17] Yao W., Tanifuji S., Takahashi K., Koyama K. (2001). Mixing efficiency in a pin mixing section for single-screw extruders. Polym. Eng. Sci..

[18] Xu B., Yu H., Turng L.S. (2018). Distributive mixing in a corotating twin screw channel using Lagrangian particle calculations. Adv. Polym. Technol..

[19] Lim K.H., Hwang W.R., Kim S.J. (2019). A finite-element technique for flows in the single screw extruder using a partial periodic unit. Korea-Aust. Rheol. J..

[20] Jian R., Dai R., Sain M., Yang W., He Y. (2022). Ductile behavior and heat transfer efficiency in polymer extrusion by self-controlled “flipping melt-pancakes” with multi-fields synergy. Int. J. Heat Mass Transf..

